# Clinicians’ Perspectives on Barriers to Discussing Infertility and Fertility Preservation With Young Women With Cancer

**DOI:** 10.1001/jamanetworkopen.2019.14511

**Published:** 2019-11-06

**Authors:** Andrea Covelli, Marcia Facey, Erin Kennedy, Christine Brezden-Masley, Abha A. Gupta, Ellen Greenblatt, Nancy N. Baxter

**Affiliations:** 1 Department of Surgery, University of Toronto, Toronto, Ontario, Canada; 2 Leslie Dan Faculty of Pharmacy, University of Toronto, Toronto, Ontario, Canada; 3 Department of Surgery, Mount Sinai Hospital, Toronto, Ontario, Canada; 4 Department of Medicine, St Michael’s Hospital, Toronto, Ontario, Canada; 5 Department of Oncology, Hospital for Sick Children, Toronto, Ontario, Canada; 6 Department of Obstetrics and Gynecology, Mount Sinai Hospital, Toronto, Ontario, Canada; 7 Department of Surgery, Li Ka Shing Knowledge Institute, St. Michael’s Hospital, Toronto, Ontario, Canada; 8 Institute of Health Policy, Management, and Evaluation, Dalla Lana School of Public Health, University of Toronto, Toronto, Ontario, Canada

## Abstract

**Importance:**

Infertility can be a devastating adverse effect of cancer treatment for young women. Fertility preservation may be an important and influential factor in treatment decisions. Despite American Society of Clinical Oncology guidelines recommending discussion around potential infertility with patients, nearly 50% of young women with cancer remain uninformed.

**Objective:**

To understand and describe from clinicians’ perspectives the barriers to discussing infertility and fertility preservation with young women with cancer.

**Design, Setting, and Participants:**

This qualitative study used purposeful, maximum variation, and snowball sampling strategies to recruit 22 clinicians from cancer centers and community hospitals in 5 Canadian provinces, 5 practice areas, and 12 practice sites. Eligibility criteria included clinicians who regularly treat young women with cancer who might need fertility preservation. Telephone interviews that lasted between 30 to 75 minutes were conducted between May and November 2014 using a semistructured interview guide. Thematic analysis was used to discern the nature of barriers, and the Cabana framework was used to organize and interpret these findings. Analysis was conducted from May 2014 until May 2015.

**Main Outcomes and Measures:**

Clinician perspectives on what influences their nonadherence to American Society of Clinical Oncology guidelines, which recommend discussing fertility preservation with patients.

**Results:**

A total of 22 clinicians were interviewed, including 8 medical oncologists, 4 surgical oncologists, 4 fertility specialists, 3 hematology and oncology specialists, and 3 nurse practitioners or clinician nurse specialists. Seventeen clinicians were women and 5 clinicians were men; the median (range) time in practice was 10 (0.67-37) years. Analysis suggested that clinicians’ unfamiliarity with infertility risks, fertility preservation technologies, referral processes, and procedures, as well as environmental factors and their perceptions of fertility preservation, influenced their practices regarding fertility discussions.

**Conclusions and Relevance:**

The findings of this qualitative study suggest that the individual and environmental challenges that clinicians experience might negatively affect their willingness and ability to raise fertility-related issues with young women with cancer. Multiple strategies are needed to address these challenges to improve overall care of young women with cancer.

## Key Points

**Question:**

What do clinicians perceive as barriers to engaging in fertility preservation discussions with young women with cancer?

**Findings:**

In this qualitative study, interviews with 22 Canadian clinicians found that they perceived knowledge, attitudes, practice environments, and patient factors influenced the likelihood of discussions regarding fertility preservation. While clinicians acknowledged the importance of fertility preservation discussions, most reported feeling unprepared to discuss it with patients with cancer.

**Meaning:**

Active strategies, such as regular and sustained interprofessional collaboration and communications that can facilitate fertility preservation education, are needed to overcome the perceived challenges to fertility conversations with young patients with cancer and to improve care.

## Teaser

This qualitative study uses interviews with 22 Canadian clinicians to examine the perceived barriers to discussing fertility and fertility preservation with young women with cancer.

## Introduction

Young women with cancer may be at risk of reduced fertility due to cancer treatment.^1^ For some, loss of ovarian function may be temporary, while for others it may be permanent.^1,2,3^ Fortunately, there have been advances in assisted reproductive technologies, including cryopreservation of oocytes and less-established options, such as ovarian tissue cryopreservation.

When facing a cancer diagnosis, women should be informed about potential fertility effects of cancer treatment and have the opportunity to discuss preservation options.^4,5,6^ To address this need, the American Society of Clinical Oncology (ASCO) released practice guidelines^7,8^ for clinicians who treat patients at risk of cancer-related infertility. These guidelines state that clinicians should “address the possibility of infertility with patients…[and] discuss possible fertility preservation options or refer appropriate and interested patients to reproductive specialists.”^7^ Despite these guidelines, an estimated 50% of women with cancer remain uninformed about the potential for cancer-related infertility,^4,5,9,10^ and even fewer are referred to fertility specialists.^11,12,13^ Given the gap between guideline recommendations and practice, we wished to explore current clinical practice to determine barriers that limit these discussions.

In this article, we report findings on the experiences and perspectives of clinicians who treat young women with cancer who might need fertility preservation. The findings were drawn from the first phase of a 3-phase study aimed at understanding fertility preservation in the context of cancer treatment by exploring the experiences of clinicians and female and male patients with cancer. Study goals included development of fertility preservation decision-making tools for patients with cancer and improving oncology care. We used data from a study of the experiences and perspectives of clinicians who regularly treat young patients with cancer to understand and describe the nature of the challenges to discussing infertility and fertility preservation. We used the Cabana framework deductively to deepen understanding of the findings and to highlight possible areas of intervention that might contribute to uptake of the ASCO guidelines. The framework identifies core factors that influence clinician nonadherence to practice guidelines.^14^

## Methods

This study was approved by the ethics review board at St. Michael’s Hospital. All participants provided verbal informed consent to be interviewed, for their interview to be audio-recorded, and for the data to be used in publications. The study results are reported in compliance with the Consolidated Criteria for Reporting Qualitative Research (COREQ) reporting guideline.

We used an interpretive qualitative research design in which clinicians’ experiences and perspectives were the starting point for understanding their nonadherence to ASCO guidelines. This perspective holds that how people act toward things is informed by the meanings they attribute to them and by the context in which they exist.^15^ Thus, it is useful for fostering deeper understanding of clinicians’ actions with respect to fertility preservation discussions in the context of cancer and factors that influence treatment patterns. Another assumption of this perspective is that meanings are multiple and are not inherent in data, rather they are discerned by the researcher or analyst.^16,17^

We used purposeful maximum variation and snowball sampling techniques^18^ to recruit clinicians in cancer centers and community hospitals in 5 Canadian provinces, 5 practice areas, and 12 practice sites. We sent email invitations to 31 clinicians in the circle of contact of the principal investigator (N.N.B.) who have firsthand experience treating young (ie, aged 15-35 years) women with cancer whose treatment might negatively affect their fertility. Participants were asked to suggest others who had the experience we were investigating and who may have been willing to participate.

A literature review that explored clinical knowledge and practices regarding infertility and fertility preservation was completed to aid in the design of the interview guide.^19^ We pilot tested the guide (Appendix in the Supplement) and revised it based on feedback from participants and the research team. The interviews examined clinical practice, patient characteristics, fertility preservation discussion practices, decision-making about fertility preservation, perspectives on fertility and oncofertility, and knowledge of oncofertility literature. A doctorate-level qualitative methodologist (M.F.) conducted individual semistructured telephone interviews that lasted between 30 to 75 minutes. The interviews were recorded to ensure accuracy of participants’ statements. Clinicians were identified by number to help ensure participant confidentiality. Data collection began in May 2014, and analysis occurred concurrently^20,21,22^ so that preliminary analyses could inform subsequent interviews. Data collection ended in November 2014, when we observed informational redundancy (ie, new participants were providing similar information to what we were discerning in our preliminary analyses).^18,20,23^ Qualitative data were analyzed from May 2014 to May 2015.

### Qualitative Analysis

The interviews were transcribed and audited to ensure accuracy of transcription and to help ensure research rigour.^24^ NVivo data management software version 10 (QSR International) was used to manage the data and facilitate analysis. Analysis included both inductive and deductive strategies. Inductive thematic analytic techniques^22,25,26,27^ followed the conventional first stage methods of qualitative analysis: sorting, organizing, and coding the data and searching for similarities and differences in and dimensions of codes and themes. A codebook was used to track ongoing changes to definitions and to ensure consistency in coding. Data were double coded in instances of discrepancies between coders. Codes and themes were inductively discerned and refined through multiple reads and comparative analyses of transcripts by 2 of us (M.F. and A.C.) and through ongoing team discussions of detailed data summaries. These data summaries included descriptive and analytic observations of the data with illustrative quotes. Themes were discerned from discussions of these summaries. Final thematic decisions were based on whether and how they addressed our research questions, whether they offered new or interesting knowledge about cancer care and fertility preservation, and whether there were enough data to thickly describe themes and attend to contradictory data.

The Cabana framework,^14^ (Figure) a widely accepted conceptual framework developed to identify key barriers to guideline implementation including knowledge (eg, lack of awareness or familiarity), attitudes (ie, lack of agreement, low outcome expectancy, limited self-efficacy,) and external environment was used deductively to understand and explain clinician nonadherence to ASCO guidelines.

**Figure.**
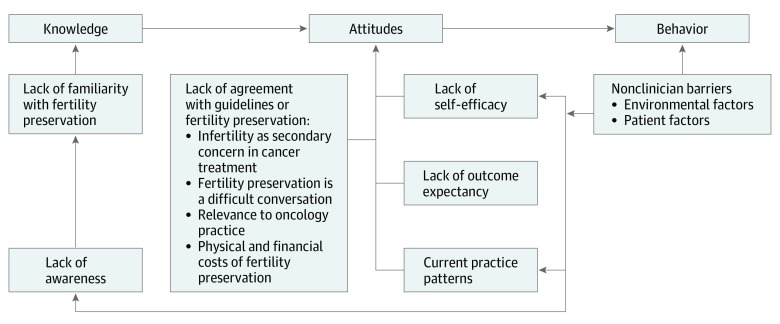
Modified Cabana Framework as Applied to the Findings

## Results

There were 22 clinicians in the final sample, including 8 medical oncologists, 4 surgical oncologists, 4 fertility specialists, 3 hematology and oncology specialists, and 3 nurse practitioners or clinician nurse specialists. There were 17 women and 5 men. The median (range) time in practice was 10 (0.67-37) years (Table).

**Table.** Characteristics of Clinicians Who Participated in Semistructured Telephone Interviews

Characteristic	No. (N = 22)
Medical specialty	
Medical oncology	8
Surgical oncology	4
Fertility	4
Hematology or oncology	3
Nurse practitioner or clinical nurse specialist	3
Sex	
Women	17
Men	5
Length of practice, y	
<5	5
5-10	8
>10	9
Median (range)	10 (0.67-37)

### Clinician Knowledge: Lack of Familiarity With Fertility Preservation

Most participants said that they were aware of ASCO guidelines, but many admitted to a general lack of familiarity with fertility preservation among clinicians (Box 1). Clinician 6, a medical oncologist, stated that oncologists were “truly poorly informed” and “remarkably ignorant about it.” Clinician 7 said clinicians were only aware of fertility “in a sort of theoretical way.” Clinician 18, a surgeon, said, “I have slightly more information [about fertility preservation] than a layperson.” Participants variously admitted that clinicians were insufficiently knowledgeable about the risks, did not understand fertility preservation processes, had no sense of timelines for how the processes work, had little or no understanding of the available technologies or their costs, and did not know where or how to refer patients for consultations.

Box 1. **Qualitative Findings and Representative Quotes Related to Clinician Knowledge**

**Lack of Familiarity With Fertility Preservation**

“Most people [oncologists] in my workplace know about oncofertility in a sort of theoretical way.” (Clinician 7)
“Some oncologists do not understand the fertility preservation process, have little or no understanding of available technologies, have inadequate knowledge and understanding of how and where to refer patients, do not have education materials at their fingertips to give to patients.” (Clinician 1)
“So I think the fertility community is aware and on board, and it’s the oncology community that still needs more work. [A major obstacle] for oncologists is just knowing what’s available and where to send patients.” (Clinician 14)
“This information gap is one that goes from oncology to patients and another from fertility specialists to oncologists. This lack of [in-depth] knowledge is one factor that prevents some practitioners from referring patients and/or raising fertility issues with them.” (Clinician 17)
“The risks of cancer treatments with patients…these risks cannot be quantified. With breast cancer-related chemotherapy for example the risk of infertility is about 50% to 60%; in colon or GI cancers they are unknown. There’s uncertainty, ‘This is the treatment I’m going to give you. I don’t know what’s going to happen to your fertility.’” (Clinician 9)
“The referring docs, I don’t think they think of it [fertility preservation] at all, …I don’t think it goes into the algorithms and the thought process at all. And then within the oncology community, I really think there’s a lot of variation among practitioners, with some people having a special interest and others who really pay no attention or pay only cursory attention. Some of them will only bring it [fertility] up if someone brings it up with them.” (Clinician 13)
“I actually did a survey in approximately 2008, and a lot of oncologists don’t know about some of the stuff we can do out there. And in fact, one of my patients went back to tell her oncologist what we were planning and he was like, ‘Well, I’ve never even heard of that. I didn’t even know you could do that, so tell Dr [name redacted] she’d better call me because I don’t understand.’ … We were going to do, she has ovarian cancer and they’re going to take out both her ovaries, so we’re going to take the eggs out of the ovaries at the time of surgery and we’re going to mature them.” (Clinician 11)

GI indicates gastrointestinal.

Even when clinicians were aware of fertility preservation, the dearth of knowledge was still evidenced in their uncertainty about links between cancer treatments and infertility, options, or risks. Clinician 17, a surgical oncologist, said, “…I don’t know in colon cancer whether the chemo is as bad for fertility or not, I don’t know.” Clinician 9, a medical oncologist, said, “…I can’t truly answer the question, ‘What is the advantage of freezing eggs vs embryos vs an ovarian transplant vs…?’ I can’t give the exact specifics.” Clinician 9 said she admits to patients that she is uncertain how cancer treatments may put them at risk of infertility, “I don’t know what’s going to happen to your fertility.” In part, clinicians’ uncertainty appeared to stem from contrary research evidence and from existing uncertainties about treatment effects. For example, clinician 16 admitted, “We can’t predict whose fertility will be impacted.” These uncertainties likely contributed to clinicians’ experiences of lack of self-efficacy.

### Clinician Attitudes

#### Lack of Self-efficacy

Clinicians’ lack of self-efficacy or the absence of confidence in their ability to appropriately and correctly engage in fertility preservation discussions with patients was partly a function of their (1) lack of preparation; and (2) perceptions of fertility preservation discussions as difficult and complex, as “really sensitive,” “awkward,” “tricky,” “a can of worms,” as clinician 7 described them, and as a “Pandora’s Box,” as clinician 21 put it (Box 2). These perceptions suggest that discussions would be difficult to have because clinicians were not conversant enough with the complex issues that typify fertility preservation discussions. Current fertility preservation evidence is characterized by contradictions and uncertainty. For example, clinician 10, a nurse practitioner, said, “There is preliminary data that suggest hormone stimulation will not precipitate the growth rates of the cancer or increase possibilities of relapse, but there is data that suggest otherwise.”

Box 2. **Qualitative Findings and Representative Quotes Related to Clinician Attitudes**

**A. Lack of Self-efficacy**

“I can’t truly answer the question, is, ‘what is the advantage of freezing eggs vs embryos vs an ovarian transplant vs…?’ I can’t, like, I can’t give the exact specifics. I know to a certain extent embryos are a bit hardier than eggs when they’re frozen, but I can’t, I don’t have that full expertise…” (Clinician 9)
“I think people realize that many of these patients are going to have long-term problems with, you know, premature menopause, difficulty having kids, et cetera. I don’t know that there is great knowledge about what the options are, just because there’ve been so few options that, just, there hasn’t been much to have knowledge about.” (Clinician 1)
“So if you know the risk [of infertility] is 100% because you took the ovaries out, that’s a no-brainer. If you know the risk is close to zero because they only got 2 drugs and neither of them damages the ovary, that’s a no-brainer too. But unfortunately, most of the time it’s in between, and then you have to make some sort of assessment of what’s the risk of infertility vs—an even more difficult assessment—what’s the risk of delaying treatment? I mean, there’s even less data on that score.” (Clinician 6)
“Discussions are difficult when patients want guarantees.” (Clinician 12)
“Fertility preservation is a Pandora’s Box, it may force patients to confront issues they may not want to discuss, ‘Do I want to trust that this relationship is permanent enough?’ ‘What if my cancer comes back?’ ‘What if I don’t survive?’” (Clinician 21)
“I think it’s a black box. they’re (clinicians) afraid to open it because they don’t have the time, generally, to stay and talk about all of the ramifications…I don’t think they maliciously avoid the topic because, you know, I think it’s simply they don’t know enough about what they could be offering or how simple it could be.” (Clinician 10)
“If they ask me, you know, you know I’ve slightly more information than a layperson on fertility issues, so I can’t be answering those questions because I might answer them wrong.” (Clinician 18)

**B. Lack of Agreement With Guidelines and Fertility Preservation**

**1. Infertility as a Secondary Concern in Cancer Treatment**

“The last thing we like as physicians is, number 1, to have something interfere with a treatment that we want to give, and number 2, to have something delay the treatment that we want to give…. I can’t even think of a situation where fertility was the primary concern, the primary concern is always...survival.” (Clinician 13)
“The last thing you want, I think, as a surgeon, is some hormone fertility therapy to delay your treatment.” (Clinician 17)
“Infertility is not the end of the world, it is not like having irreversible heart disease or another tumor, and there are other avenues to parenthood. The perception of infertility as an unnatural phenomenon is rooted in the understanding that it is a known toxicity of cancer therapies. Outside its causal relationship to cancer it is perceived as a natural occurrence.” (Clinician 5)
“Although it [fertility] is an important issue it is not life-threatening, it’s not like the life and death threat of cancer.” (Clinician 6)
“When you’re faced with a patient that has cancer that needs treatment…worrying about preservation is secondary. For males, it’s probably a lot easier to go to sperm bank, for females it seems hard to go and egg-harvest.” (Clinician 12)

**2. Fertility Preservation as a Difficult Conversation**

“Overwhelming them [patients] with additional information about fertility preservation, particularly when patients are in shock on learning they have cancer and when multiple treatments are required.” (Clinician 9)
“We’re asking young women to make decisions about something that may or may not be a problem. And not only that, we’re asking them to pay for it, and at an emotionally charged time in their lives, and it may incur a delay in treatment because they go see the fertility expert, they want to wait a cycle to harvest or so forth …it’s another thing they have to deal with at a really high-pressure time.” (Clinician 16)

**3. Relevance to Oncology Practice**

“I know surgeons who see patients who don’t even know if they’re going to need chemo, and you won’t know because you don’t have the pathology until you take the whole tumor out, and then it’s almost like, ‘Well, we’ll get to that problem if we need to,’ because they may not need chemo, and they may not need this and they may not need that, and so their fertility may not be affected, so why start this whole talk for nothing, right?” (Clinician 17)
“Doctors don’t understand just how urgent this is, first of all how fast some of these lymphomas can progress so we need to get them going [receiving treatment] very quickly.” (Clinician 13)
“For aggressive cancers…well, a woman cannot have a baby if she is dead.” (Clinician 5)
“Not knowing in advance of surgery that chemotherapy will be needed or only finding out after surgery means women are not referred for fertility consultations early enough.” (Clinician 14)

**4. Physical and Financial Costs of Fertility Preservation**

“Physical recovery might be difficult because of risks related to fertility preservation; eg, endocrine-related side effects and hyper ovarian stimulation syndrome, which causes pain and discomfort.” (Clinician 21)
“From a psychological standpoint, it [fertility preservation] does add another stress because you are adding a whole other element of treatment because you have to take the medications to stimulate your ovaries, you have to go to the procedure to harvest them, and then after that then we then hit you with your chemotherapy. Like, it’s a lot physically and mentally for someone to endure, like even each on their own is actually not easy, and now you’ve got both of them together.” (Clinician 9)

**C. Lack of Outcome Expectancy**

“There are women for whom, you know, waiting a few weeks to do fertility preservation could compromise their cure rate. So there are these women who have something called *triple-negative breast cancer*, which seems to be a more rapidly progressive kind of breast cancer, so could those few weeks make a difference? And the literature is contradictory, some suggest yes, some suggest no.” (Clinician 7)
“We can’t guarantee that that’s not going to happen if they (A) delay their treatment or (B) if they have a hormone-sensitive tumor. We can’t say definitively that an IVF cycle doesn’t increase their risk. It doesn’t appear to, research doesn’t show that it does, but we can’t say 100%.” (Clinician 10)
“I guess what’s hard to find is the true success rate of making babies from frozen eggs, number 1; number 2, you know, which fertility bank is the best bank to send patients to for egg freezing. Like, embryo freezing is pretty standard, fine, but the concern is for women who don’t have partners and they want to just freeze their eggs, you know, should they go, where should they go, because it’s a big cost and it’s a big deal to refer people without knowing.” (Clinician 2)
“You’re paying for an unknown return on investment… with most fertility centers, they’re going to underwrite everything and not make any promises that anything’s going to work out, right, and then you’ve expensed x thousands of dollars, you’ve delayed your care and treatment for a hope… It’s not like, ‘Oh, yeah, here you have a 40% chance if you subscribe to this menu, this recipe, and sign here, and pay,’ right? If you have a 40% chance, at least they sort of know. But because of the nature of fertility, it’s a lot of unknown, a lot of hope.” (Clinician 3)
“Although egg cryopreservation is good, the thawing process is still unreliable.” (Clinician 21)

IVF indicates in vitro fertilization.

Some surgeons expressed fears about being asked fertility-related questions because they “might answer them wrong,” as clinician 18 put it, or “give them inaccurate information,” as clinician 17 put it. Clinician 6 expressed concerns about engaging in “informed guesswork.” The clinicians’ experience of lack of self-efficacy would likely be heightened in situations in which patients were fairly well informed but were seeking additional information or advice. Clinicians who did not regularly treat young patients with cancer were particularly likely to feel unprepared and not raise fertility issues. According to clinician 12, a hematologist, “Unless you’re doing a young patient…or that’s your typical practice, it might fall off the radar, and then the less you do it, the more uncomfortable you become, the more you might forget it.”

#### Lack of Agreement With ASCO Guidelines and Fertility Preservation

Participants all agreed in principle with the ASCO guidelines, but they also voiced skepticism about fertility preservation and its relevance to their practices. Some clinicians’ comments suggested adherence to guidelines was impractical and reduced their autonomy. Discussing fertility preservation might be inappropriate in cases of very young or older patients, when patients have aggressive forms of cancer, or when mortality is imminent. In such cases, clinician 19 suggested fertility was a “luxury.” Also, clinicians’ perspectives and characterizations of fertility preservation and infertility suggested they did not fully support or believe in its applicability to their practice. Clinicians conceived of infertility as nonfatal and therefore fertility preservation was secondary to cancer treatments. As clinician 5 explained, “A woman cannot have a baby if she’s dead.” Clinician 6, a hematologist, contended that infertility was not the worst possible scenario, that it was not “like having irreversible heart disease or another tumor.”

Clinicians’ lack of agreement was also suggested in the characterization of fertility preservation as a burden on both clinicians and patients. Clinician 6, a surgeon, said it was “one more thing to deal with,” and clinician 9, a medical oncologist, said a cancer diagnosis was traumatic for patients and discussing fertility preservation added another layer of distress. Clinician 9 went on to explain, “Even at the best times, when you don’t have cancer and you’re trying to figure out fertility and family planning, it’s not easy.” These discussions also added to clinicians’ workload, particularly when they were unfamiliar with it; for example, clinician 13, a hematologist, admitted, “We hate being asked questions that we know nothing about because it means that we have to figure it out and spend time outside of the clinic to go and find out, and figure out, and call up, and stuff.” Other clinicians perceived fertility preservation as a complication that might negatively affect treatment outcomes or interfere with treatment, ie, the time required for fertility preservation may lead to delays or potentially worse oncologic outcomes. Such concerns were suggested in the comments of clinician 17, a surgical oncologist, who said, “The last thing you want, I think as a surgeon, is some hormone therapy to delay your treatment” and in the comments of clinician 12, a hematologist, who asked, “How long is it going to take to get this person seen and get done whatever needs to be done so I can get on with treatment?”

Clinicians also suggested fertility preservation was a financial burden with ethical undertones because it is costly and, as clinician 16, a surgical oncologist, observed, “We’re asking them to pay for it at an emotionally charged time in their lives.” Indeed, clinician 19, a fertility specialist, asserted that fertility preservation was both a “new and untested science [and] a private, for-profit venture.” Clinicians’ concerns about the physical risks involved (eg, pain, discomfort, effect on cancer cure rates) and potential psychosocial burdens of undergoing fertility preservation all suggested lack of agreement with it.

#### Lack of Outcome Expectancy

Clinicians’ lack of outcome expectancy or the expectation or belief that engaging patients in fertility preservation discussions and referring them for preservation would not result in successful outcomes was suggested in their negative assessments of and reservations about fertility preservation evidence, technologies, benefits, and successes in the oncologic context. Clinician 13, an oncologist, noted that, outside of embryo cryopreservation, the evidence regarding the success of preservation had “not been great,” was “contradictory,” and that outcomes were largely unknown and difficult to quantify. Others pointed to the experimental nature of technologies, such as ovarian tissue preservation, which had success rates that clinician 11, a fertility specialist, described as “okay,” and in vitro maturation, which had success rates that clinician 14 characterized as “not great.” Perhaps just as important, clinicians’ lack of outcome expectancy was based on their awareness of successful birth rates among healthy women and their perceptions that fertility preservation in the context of cancer was likely to be less successful. They maintained that fertility preservation outcomes in healthy women could not be readily extrapolated to the cancer context because, as clinician 10 put it, healthy women have “tons of eggs; [donors] are young and do not have cancer.”

### Current Practice Patterns

Clinicians’ descriptions of current practices about fertility preservation were characterized by contrary perspectives about fertility preservation and attributions of responsibility for it to others (Box 3). Although clinicians acknowledged that infertility was important and a significant adverse effect of cancer treatments, they also asserted that most clinicians were on board with fertility preservation in theory only; in other words, if they do not have responsibility for it. They also agreed that there were variations in perspectives and approaches to fertility preservation among practitioners. Clinician 13, a hematologist said, “Some people have a special interest, and others really pay no or only cursory attention,” but overall many clinicians overlooked fertility entirely. Clinician 13 went on to say “I don’t think they think of it at all, …I don’t think it goes into the algorithms and the thought process at all.” Moreover, clinicians admitted that some oncology clinicians tended to focus only on the part of the body they are treating and did not see preservation as their responsibility. Clinician 8 reported hearing from colleagues that “I’m not giving them chemo, why should I do the referral?” and clinician 11 recounted hearing a colleague say, “It’s not my problem…I don’t destroy the ovary.”

Box 3. **Qualitative Findings and Representative Quotes Related to Current Practice Patterns and Nonclinician Barriers**

**Current Practice Patterns**

“The failure of oncologists to engage with fertility is truly a failure because it is not a lack of available information or knowledge, it’s more the physicians’ lack of interest, lack of accessing available information, lack of acting on it, and a lack of patience to actually take the initiative.” (Clinician 6)
“Oh, yeah, I heard about this. That’s a problem, isn’t it? But I don’t know where to send them, yet there are multiple fertility preservation clinics in [urban Canadian city].” (Clinician 6)
“It’s [fertility] an important thing that’s overlooked, especially in oncology care.” (Clinician 12)
“Although infertility is a big-time side effect of cancer treatments or a huge component of treating patients, fertility preservation does not tend to be high on the priority list of some cancer-treating specialists.” (Clinician 20)
“Some [clinicians] do not see the relevance of [in]fertility to their practice, others tend to focus on their own scope of practice, on their part of the body, and still others do not see fertility preservation as being their concern, *‘*Well I’m not giving them chemo, why should I do the referral?’ or ‘It’s not my problem,… I don’t destroy the ovary.’” (Clinician 11)
“Patients that report ‘nobody told me’ are just too many to list. For every 1 patient who is sent [to a fertility specialist], I’m sure there are 10 or 20 who were diagnosed but weren’t offered the option to see a fertility specialist. The word just doesn’t feel like it’s getting out to all of oncology. How [can] a treatment that can compromise fertility so severely not be discussed?” (Clinician 10)

**Nonclinician Barriers**

**A. Environmental Factors**

“Sometimes doctors just don’t have the luxury of time or resources to be able to assure them that they are going to remain fertile.” (12)
“My center does not even have pamphlets/reading materials to give to patients, I think that’s how bad we are at it, just not even hitting the radar, really.” (Clinician 12)
“When they come from afar sometimes they aren’t [offered fertility preservation]. Sometimes the physician may not have access to that in their community so they don’t offer it as an option, but once they’re in [urban Canadian city], we really kind of mobilize and say, you know, ‘You need a second opinion because we can offer you that service in this city.’” (Clinician 10)
“Preservation options are sometimes not available, there is no place to send patients for preservation procedures, or that options are limited to medications such as birth control pills.” (Clinician 16)

**B. Patient Factors**

“You cannot make an assumption about finances. You have to offer the service, and then it’s up to the patient to decide if they can afford it. But I think… some of these people feel that there’s no way, because she works at Tim Hortons, that she can afford this. Sometimes we’ll even see in the referral… it’ll say something like, ‘Not a financially feasible option, but she wanted to see you anyway,’ type of thing, You’re kind of like, ‘Okay, so the people who weren’t pushing and maybe didn’t seem like they had the money, they probably weren’t referred at all.’” (Clinician 10)
“Although costs for male fertility preservation is largely nominal, it is not the case for women, whose processes are more complex. The costs are a game changer for many women.” (Clinician 15)
“The costs are not completely covered, and many patients, such as students and new immigrants, are still unable to afford it.” (Clinician 8)
“Patients might not have easy access to fertility clinics because of their geographic location. In these instances, they may have to travel long distances to gain access, which often means travel costs are added to the fertility preservation-related ones.” (Clinician 15)
“They might want children 10 years down the line…they can’t think that far into the future and see the cancer in front of them, they don’t really want to have to think about the other aspects.” (Clinician 9)

Clinician 9, a medical oncologist, admitted that they are the entry point into fertility preservation discussions but maintained that it was really the purview of “true fertility people”(ie, fertility specialists). One such specialist, clinician 10, stated that the number of times “patients report that ‘nobody told me about fertility preservation,’ are just too many to list. For every 1 patient who is referred, there are many more who are not.” Although some clinicians said they had agreed within their multidisciplinary teams that whoever meets with the patient first should introduce the topic of fertility issues and although fertility specialists and oncologists have formed partnerships to develop educational programs for oncologists, overall, participants admitted that there was very little interprofessional collaboration or communication between fertility specialists and oncologists.

### Nonclinician Barriers

#### Environmental Factors

Environmental factors, such as the organization of care (eg, timing of diagnoses and tests), lack of time and resources (eg, support staff, nurses), structural supports (eg, referral systems), and patient education were also challenges to fertility preservation and discussions. Clinicians noted that it was difficult to provide patients with risk information or advise them when there was uncertainty about diagnoses and treatment plans. Clinicians said they might learn late in the diagnosis-treatment continuum that a patient needs chemotherapy, and if the cancer is aggressive, there might not be time for fertility preservation. In such instances, clinicians said they might not introduce the topic. Likewise, clinicians said that when a treatment plan was already in place, it was very difficult to pause the process by introducing the issue of fertility.

Clinicians stated that there was already insufficient time for oncology consultations, which can be complex and lengthy, and thus there was not enough time to discuss fertility preservation. They also pointed to insufficient resources, such as nurses or social workers, who could act as backup for fertility discussions and referrals. Fertility discussions and referrals may not happen when clinicians must navigate the system themselves or when they work in geographic locations where access to fertility preservation options are limited or referral systems were not well established. Referrals were also less likely to happen when clinicians had no relationships with fertility clinics.

#### Patient Factors

Along with the nature of diagnoses, mortality concerns (eg, aggressiveness of cancer, incurable malignancies), psychosocial considerations (eg, patient anxiety, stress, and information overload), and clinicians’ perceptions of patients’ status (eg, financial position) were also factors that informed fertility discussions. Clinician 4 expressed concerns that patients were “already very stressed [and] were getting a lot of information at a time when they’re already overwhelmed with this diagnosis.” Many clinicians voiced concerns about affordability of fertility options for patients. Clinician 15, a nurse practitioner, observed that “the costs are a game changer for many women,” and clinician 8 noted that “many patients, such as students and new immigrants, are still unable to afford it.” Perceptions about patients’ socioeconomic status also appeared to influence clinicians’ willingness to initiate fertility preservation discussions. About their colleagues, clinician 10 said, “Some of these people feel that there’s no way, because she works at Tim Hortons, that she can afford this. Sometimes we’ll even see [that] in the referral.”

## Discussion

In this qualitative study, we used data from a study of the experiences and perspectives of oncology practitioners to describe factors that influence their nonadherence to ASCO guidelines. We found that unfamiliarity with fertility preservation processes and risks, lack of agreement with guidelines and fertility preservation, perceptions of infertility and fertility preservation, lack of confidence in their abilities, uncertainty about successful outcomes, environmental considerations, deficient referral systems, and patient-related factors all contributed to nonadherence.

Our finding of a connection between outcome uncertainty and discussions of fertility is reaffirmed in existing studies that found that the likelihood of success of preservation techniques and perceived poor oncologic prognosis were deterrents to fertility discussions.^28,29,30^ Another finding of the study is the reluctance among clinicians to engage in conversations that may negatively affect patient survival. This hesitancy is likely a function of their perceptions of infertility as nonfatal, beliefs about the urgency and importance of timely oncologic treatment, the potential fatality of cancer, and the desire to protect patients from potential harms. It is also likely a consequence of their perceptions of fertility preservation as a burden, a complication, and a secondary and often opposing objective. Not initiating fertility discussions challenges the principles of patient-centered care and, in the context of cancer, suggests that clinicians in this study may be overestimating the importance of survival outcomes while underappreciating the psychosocial concerns of patients with cancer beyond that of survival, namely potential future infertility^31,32,33^ and quality of life.

Our findings also suggest a siloing in practice whereby clinicians do not see the relevance of fertility preservation to their practices and feel that these discussions are not their responsibility because their treatments do not cause infertility. This finding suggests insufficient interprofessional education and collaborations (eg, between oncologists and reproductive specialists). Such lack of communication likely contributes to clinicians’ lack of awareness of fertility preservation and referral processes, a finding that has been reported in several studies.^34,35,36^

Using the Cabana framework^14^ in this study helped to highlight how knowledge around treatment options and outcomes, beliefs about oncology care, and limited access to fertility specialists and referral resources may have interacted to negatively affect oncology specialists’ engagement in fertility preservation discussions. The findings suggest that knowledge and attitudes are closely aligned with behavior and reaffirm research that found that modifying knowledge and attitudes is fundamental to successful adoption and implementation of clinical practice changes.^14,33,37,38^ These findings also suggest that knowledge translation strategies, such as galvanizing local opinion leaders and society endorsements, conducting educational meetings, and continuing medical education opportunities,^37,39^ are potential solutions to improving clinicians’ competencies, reorienting attitudes, and improving patient care. Such approaches could operate in tandem with patient education and development of support resources and patient navigation tools to help young adults initiate fertility discussions.

### Strengths and Limitations

Our study had some strengths and limitations. Although we used maximum variation sampling in our study, qualitative research sampling is purposeful rather than random, thus findings are not generalizable in the statistical sense. The study design facilitated in-depth insights into the barriers to fertility preservation discussions, and the use of a conceptual framework increased the transferability of the findings to other contexts. The findings reflect and expand on what has previously been described for individual treatment centers, single disease sites, and other health care infrastructure,^28,30,34,35^ demonstrating that the barriers we described have relevance for other treatment sites, health care systems, and oncologic diagnoses. A potential limitation of this study is self-selection bias because clinicians who chose to participate may have had more interest in fertility preservation than clinicians who chose not to participate.

## Conclusions

The findings in this qualitative study suggest that although fertility preservation discussions have been a long-standing standard of care, in many instances they are not standard care in oncology. Our findings also suggest that medical education has not kept pace with fertility preservation technologies, which has left many clinicians uninformed about them. Moreover, awareness of the ASCO guidelines has not translated into increased discussions about fertility preservation. Active knowledge translation strategies, such as interprofessional collaborations and communications, identification of local opinion leaders or champions, society endorsements, continuing medical education opportunities, dedicated fertility preservation programs, referral networks, and decision-support systems, are needed. Creating resource tools (eg, posters, decision aids) for clinicians and patients also has potential to improve levels of fertility-related discussions.

## Notes

Supplement. **eAppendix.** Interview Guide
