# Supplementary material for: Clinicians’ Perspectives on Barriers to Discussing Infertility and Fertility Preservation With Young Women With Cancer
Source: JAMA Netw Open. 2019 Nov 6;2(11):e1914511. doi: 10.1001/jamanetworkopen.2019.14511 (PMC6865261; doi:10.1001/jamanetworkopen.2019.14511)
Supplement: Supplement. — eAppendix. Interview Guide [file jamanetwopen-2-e1914511-s001.pdf]

Supplementary Online Content

Covelli A, Facey M, Kennedy E, et al. Clinicians' perspectives on barriers to discussing infertility and fertility preservation with young women with cancer. *JAMA Netw Open*. 2019;2(11):e1914511. doi:10.1001/jamanetworkopen.2019.14511

eAppendix. Interview Guide

This supplementary material has been provided by the authors to give readers additional information about their work.

© 2019 Covelli A et al. *JAMA Network Open.*

eAppendix. Interview Guide.
*Thank you for participating in this study. This interview will last between 30 to 45 minutes. I will ask you about your perspectives on fertility preservation and about your experiences with treating adult and young adolescent women who are newly diagnosed or have been previously diagnosed with cancer. I will start by asking you about yourself, your training, and your practice. This interview is confidential and if we use data from your interview in publications, we will take steps to protect your identity. Do you have any questions before we begin? *(Ask participants if they have done a QR interview before, if no briefly describe how the process works).*

A. Clinical Practice
   1. Tell me a little about the type of clinical setting in which you work
   2. In your role, what do you do?
   3. How long have you been in this role?
   4. Are you early, mid, or late career?
   5. In your role, where are you in the cancer diagnosis –treatment- fertility preservation regime?
      - At what point in this process would you see a patient who would need fertility preservation referral/consultation

B. Patient characteristics
   1. Tell me a little about your patients
      - Types of patients you generally see in your practice?
      - Who are they, generally (age, gender, etc.)
      - Types of cancer

C. Fertility Preservation Practices/Approaches
   1. How often do you encounter situations where you discuss fertility preservation with patients or their families?

   2. Who are the patients with whom you generally have this discussion?
      - Based on age, diagnosis, treatment type?

   3. Is there a best time to discuss options for fertility preservation with patients? If so, when?

   4. How do you decide if you will raise a discussion about fertility preservation?
      - What usually prompts you to have these discussions?

   5. Can you describe in as much detail as possible what happens in these discussions?
      (or, think about a situation when you've had this discussion with a patient…..
      - What sorts of things do you discuss?
      - What fertility preservation options?
      - All options available; e.g., embryo, oocyte and ovarian strip preservation; medications that protect fertility?
      - Risks and benefits of each option?
      - Have there been situations when did not discuss all options? Please describe (e.g. why not?)
      - Have you had situations where you've discussed risks (e.g., premature ovarian failure) with women after they have undergone their treatments? If yes, describe.

   6. Can you recall and describe for me a situation when a discussion about FP was particularly challenging
      - (What made it difficult – systems, interpersonal, etc.)

© 2019 Covelli A et al. *JAMA Network Open.*

7. How do you think other doctors regard fertility preservation among AYAs?

8. What are your thoughts on it, specifically among AYAs?

D. Barriers and Facilitators
   1. What concerns do you have with respect to fertility preservation in your practice?
   2. What are some of the challenges that come up in your practice?
      - What makes it difficult for you to treat your patients?
      - What makes it easy?
      - What makes it difficult for you to talk about fertility with your patients?

E. Perspectives on Patient Decision-Making
   1. What do you think makes it difficult for patients to decide about fertility preservation?
      - What are they usually most/least concerned about? e.g., their priorities
      - What do you think is most/least important to them when deciding between preservation options?
      - What are you usually most/least concerned about?

   2. What do you do when patients express concerns about fertility preservation?
   3. What are some things that have led to patients changing their decisions after talking with you?
   4. Are there any decision-making aids available that you use with patients?
      - Yes/no. Please describe
   5. What do you think about how information (e.g, about options) is presented to women?
      - Are there other ways to this present information? If yes, how?

F. Perspectives on(onco)fertility literature/knowledge
   1. What are you usually reading in terms of the literature in this area?
      - Do you keep up with research on the topic of oncofertility in AYAs?
   2. What are your thoughts about state of current research on oncofertility?
      - gaps in knowledge/understanding?
      - where we need to go with research, training?
      - where we're at in our understanding of the issues?
      - biggest concerns about the literature/research in this area
      - Adequacy in terms of what it offers health care providers re guidance on patient referral protocol; e.g., when and how to refer?

Is there anything you think we should know that I haven't asked you about?

Are there any other health care providers that you can think of that we should interview about this topic? (Names and practice locations)?

© 2019 Covelli A et al. *JAMA Network Open.*
